# Prioritizing risk factors and identifying target areas to address with interventions to improve sustainable employment of persons with a brain injury or a spinal cord injury – A multi-stakeholder consensus process

**DOI:** 10.3389/fresc.2023.1049182

**Published:** 2023-02-17

**Authors:** Monika E. Finger, Katarzyna Karcz, Barbara Schiffmann, Julia Eugster, Melissa Selb

**Affiliations:** ^1^ Work and Integration Unit, Swiss Paraplegic Research, Nottwil, Switzerland; ^2^ Department of Health Sciences and Medicine, University of Lucerne, Lucerne, Switzerland; ^3^ Work and Integration Unit, FRAGILE Suisse, Zurich, Switzerland

**Keywords:** sustained employment, spinal cord injury, acquired brain injury, consensus process, multistakeholder approach

## Abstract

**Background:**

Achieving sustainable long-term employment is the goal of work integration for persons with acquired brain injury (ABI) or spinal cord injury (SCI). However, decreasing employment rates over time for persons with ABI and SCI indicate that remaining employed in the long-term is a challenge.

**Purpose:**

To identify the most important risk factors that pose a barrier to sustainable employment of persons with ABI or SCI from a multi-stakeholder perspective, and to propose corresponding interventions that address them.

**Methods:**

Multi-stakeholder consensus conference and follow-up survey.

**Results:**

From 31 risk factors to sustainable employment of persons with ABI or SCI identified in previous studies, nine were defined as most important to address with interventions. These risk factors either impacted the person, the work environment or service provision. Potential interventions to address these factors were proposed in mixed condition groups, of which ten were voted on as priority interventions. The follow-up survey revealed strong agreement on the intervention proposals, strong to moderate agreement on impact, but moderate to low feasibility, as most of the interventions were measures at the meso- (service) and macro- (legislation and state regulation) level.

**Conclusions:**

Holding micro-level stakeholder conferences is a valuable method for identifying the most important risk factors to sustainable employment and for developing measures to address them. To implement measures that involve decisions at the meso- or macro-level, representatives from these levels of the healthcare and social system have to be involved.

## 1. Introduction

Being engaged in sustainable, remunerative employment is an essential part of social participation for most adults in industrial countries (1). This is also true for persons living with a disability, e.g., persons with acquired brain injury (ABI) or spinal cord injury (SCI) (2, 3).

Both ABI and SCI are health conditions known for their high, long-term global burden with far-reaching physical, emotional, and economic consequences for the affected person, their families, and society at large (4). ABI affects the brain, and depending on the severity of the injury, the persons may experience very different consequences. While physical consequences may include paralysis, sensory damage or speech and language disorders, research shows that over 80% of people with brain injuries show no or only minor visible symptoms after rehabilitation (5). Cognitive consequences are often referred to as “invisible problems”. They can relate to memory and attention disorders, reduced mental resilience and increased fatiguability, as well as perception or behavioral problems. In the absence of motor symptoms, the consequences of invisible disorders often only become apparent when the person with ABI experiences stress at home and at work (6–8). Persons with SCI, on the other hand, mostly experience mobility-related impairments, impacting on activities of daily living, toileting and transportation (9, 10). They may also suffer from problems such as incontinence, susceptibility to pressure sores and respiratory infections and pain (11–13).

Supporting persons with a disability to return to suitable work has been identified as an important field of action by vocational experts, researchers and increasingly also by the social security system and politics in many countries (14–16). High employment rates not only provide financial relief to the social system, being sustainably employed has also shown to directly benefit persons with disability, specifically sustainable employment has shown to be a strong predictor of social participation, better health, general well-being and economic success in both diagnostic groups (ABI and SCI) (17–19).

In Switzerland, the move towards strengthening vocational rehabilitation and work integration of persons with disability is reflected in the increasing number and quality of integration measures and also in legislation that now finances early intervention and long-term measures for work integration (20, 21). However, information on the employment situation of the disabled worker after the vocational rehabilitation process has ended, is scarce. There are no official national or insurance databases that contain accessible information on work status, disability status and health over the life course of persons with a disability. There is, however, quantitative evidence on long-term employment from a Swiss-wide cohort study called “SwiSCI”, that was initiated by the SCI patient organization in 2008 (22). There is also information provided by the counseling services of SCI and ABI patient organizations (23, 24). Both services report an increasing rate of workers with SCI or ABI who drop out from work some years after a successful return-to-work. This finding is in line with findings from longitudinal studies from the United States (25, 26).

To identify the factors that support sustainable employment or lead to premature drop-out of persons with ABI or SCI and to gain a deeper understanding of the mechanism and interplay of identified factors in Switzerland, we conducted a first project that included six qualitative studies (focus groups and semi-structured interviews) and two separate scoping reviews on facilitators and barriers impacting long-term employment for persons with ABI and SCI, respectively (27, 28). The scoping reviews included quantitative and qualitative studies. The quantitative studies included in the SCI review showed that time since injury, age and education are important. This was also found in the ABI review. The ABI review additionally identified severity of initial injury, chronological age, age at injury, and pre- injury education as predictive for working long-term. The qualitative studies in the SCI review highlighted the positive factors self-advocacy, self-managing health behaviors, and supportive work environment, while the barriers were related to time organization, societal attitudes and health-related symptoms such as infections, decubiti, and pain, as well as an age-related decrease in mobility. The factors in the ABI studies mostly addressed work maintenance and the key role of cognitive problems, such as slower thought processes, concentration problems, and increased fatigability with slower recovery rate. The ABI studies also emphasized the importance of having adequate coping strategies and the positive impact of flexible work schedules and supportive colleagues.

The qualitative studies evaluated the perspectives of injured workers with ABI or SCI, health, work, or insurance professionals, and employers of persons with ABI or SCI (29–31). The qualitative study results showed that the most influential facilitators for sustained employment from the injured person's perspective are adaptation of the workplace and work schedule, a supportive social environment at home and at work, and ability to self-advocate, communicate and cope with a disability. The latter is consistent with the employer's claim that good self-management combined with proactively communicating needs are important prerequisites for long-term employment. Employers also emphasized the importance of congruency between the job performance expectations of the employer and the employee while the participating health professionals highlighted the need for high quality of services and education of work integration professionals and insurance representatives on the respective condition.

These studies provided us with a more detailed picture of the work reality of person with ABI and SCI in Switzerland, revealing 31 risk factors that were able to be grouped into six main target areas. Exploring the work participation of both diagnostic groups enabled us to identify similarities that provide some insight in living with a disability in Switzerland. In contrast, the differences suggest that general knowledge about disability is not enough but rather the professionals involved also require specialized knowledge about the consequences of the particular disease.

The results of the preliminary studies identified target areas and factors that facilitate or hinder sustainable employment of individuals with SCI or ABI in Switzerland. However, it is important to understand the specific impact that each factor has on sustained employment in order to develop targeted solutions that enable persons with SCI or ABI to successfully remain in the labor market. A prioritized list of risk factors to be addressed is needed to enable feasible implementation of such interventions, especially given limited human and financial resources.

### 1.1. Research aim

To identify the most important risk factors that pose a barrier to sustainable employment of persons with ABI or SCI from a multi-stakeholder perspective, and to formulate corresponding interventions that address them.

Specific aims are:

(1) to prioritize the previously identified risk factors that pose a barrier to sustainable employment of persons with ABI or SCI.
(2) to formulate corresponding interventions for the prioritized risk factors.
(3) to evaluate feasibility and impact of the corresponding interventions.
(4) to discuss similarities and differences between ABI and SCI regarding long-term sustainable employment.

## 2. Methods

### 2.1. Overview of the process

**Figure 1:**
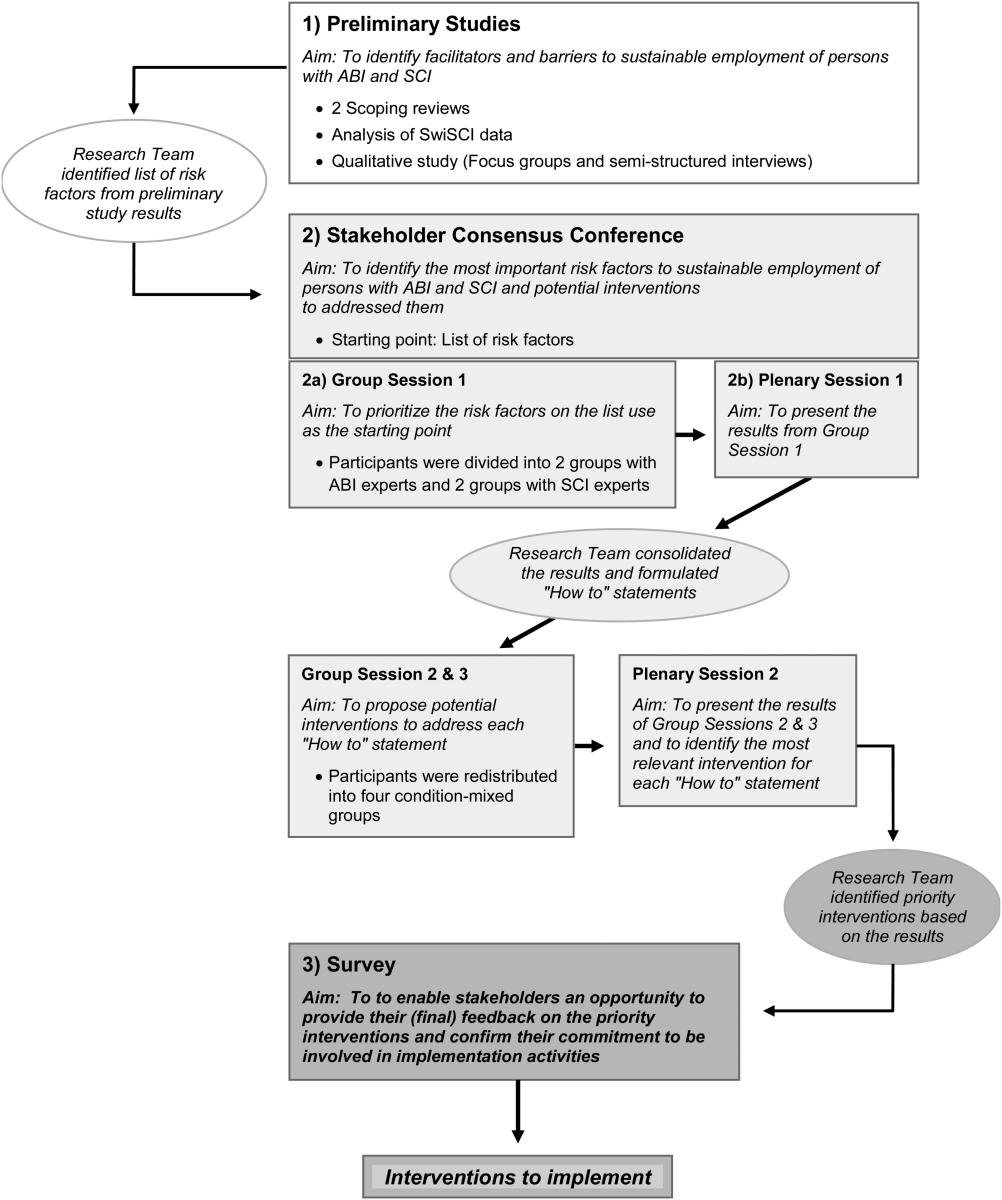
Consensus process.

This study employed a multistage decision-making and consensus process approach in line with the so-called “Q-methodology”, including a stakeholder conference and a follow-up evaluation survey (32). The Q-methodology employs qualitative and quantitative techniques to capture individual viewpoints of participants, involving the ranking of statements. See Figure 1.

### 2.2. Stakeholder consensus conference

A stakeholder consensus conference took place on 3 September 2021 in Olten, Switzerland. The conference aimed to decide the most important risk factors to sustainable employment of persons with ABI and SCI and potential interventions that address these risk factors. In addition, the research team organized the conference so as to foster knowledge exchange among the participants (patients, employers and professionals), especially to promote the exchange between participants with expertise in ABI and SCI.

Participants had expertise in ABI and/or SCI and were either persons with ABI or SCI, employers of persons with ABI or SCI, service providers, such as health- and work integration professionals, or representatives from the patient organizations and from accident and disability insurances. Potential participants were identified and invited based on contact lists from the six preliminary qualitative studies, supplemented by recommendations from experts in the field and an internet search to cover the whole spectrum of knowledge and experiences on employment of persons with SCI or ABI (see Table 1). After acceptance, the participants received detailed information on the results of the preliminary studies to become familiar with the conference content.

**Table 1:** Participants of the consensus process (conference, survey) and preliminary studies.

Perspective/Function	Participants of		
	Conference	Survey	Preliminary studies
Person with ABI	X	X	
Person with ABI	X	X	X
Professional- ABI; Head of outpatient vocational rehabilitation institution	X	X	
Person with SCI	X	X	X
Professional; Diversity Management, insurance company	X	X	
Employer- ABI	X	0	X
Person with ABI	X	X	X
Professional- ABI/SCI; Head of work integration, Disability insurance	X	X	
Professional- SCI/ABI; Case Manager accident insurance	X	X	X
Professional- ABI: NGO, Head of district, Vocational integration-ABI	X	0	
Professional/Employer- ABI/SCI; Medical director, rehabilitation center	X	0	
Professional- SCI; Patient organization SCI, support	X	0	
Professional- SCI; Medical Director in-patient rehabilitation	X	X	
Professional- ABI; Occupational therapist, Vocational integration specialist, in-patient rehabilitation	X	0	X
Person with SCI/Professional-SCI; Patient organization	X	X	
Professional- ABI; Case Manager, Disability insurance	X	X	
Professional- SCI; Patient organization, support	X	X	
Professional- SCI; Case Manager, Disability insurance	X	X	
Professional- SCI: Job Coach Vocational rehabilitation and integration	X	X	
Professional- SCI; Job coach, Vocational rehabilitation and integration	X	X	
Employer- ABI/Professional; Expert social insurance	X	X	X
Professional- ABI; Patient organization	X	X	
Professional- ABI; Patient organization	X	X	
Professional- ABI: Director vocational integration and coordination project	X	X	X
Professional- SCI: Head of social welfare department	X	0	X
Professional- SCI: Patient organization, support	X	X	
Person with ABI	X	X	X
Professional; Researcher, Work and health, supported employment, university of applied sciences North-Western Switzerland	X	X	
Professional- ABI; Neurologist	X	X	X
Employer- SCI*		X	X
Professional; NGO; Vocational integration for persons with disability*		X	X
Employer- ABI*		X	X
Employer- SCI*		X	X
Professional-ABI; Physician, consultant patient organization*		X	
Person with SCI/Professional- SCI; patient organization*		X	X
Employer- ABI*		X	X
Employer- SCI*		X	
Professional; NGO; Vocational integration for persons with disability*		X	

Abbreviation: ABI, Acquired brain injury; SCI, Spinal cord injury; NGO, Non-governmental organization.

* Persons unable to attend the conference but were interested in being involved.

The conference procedure involved a multi-stage-process with alternating group and plenary sessions (see Figure 1). The conference was facilitated by a moderator who was familiar with work integration, sustainable employment, ABI and SCI. At the start of the conference, the moderator introduced the participants to the aims of the conference, and the research team presented the risk factors identified in the preliminary studies.

The participants were then divided into four groups – two comprising participants with experience on in ABI and two groups comprising participants with experience in SCI. This group distribution was chosen to ensure a balance of the risk factors identified for ABI and for SCI. In group session 1, the participants in each group discussed the list of 31 risk factors for sustainable employment identified in the preliminary studies. The factors were grouped into six target areas: (1) rehabilitation and integration, (2) the injured worker and its social environment, (3) work environment, (4) work capacity, (5) social insurances and payers, (6) institutional and professional support. Participants had the opportunity to add new factors they felt were missing (see the list of risk factors in Supplementary Appendix S1). Lastly, each participant was asked to vote on the four factors he or she considered the most important for sustainable employment from the point of view of the target diagnostic group and from his or her personal experience.

The research team decided before the conference started that no more than eight or nine risk factors would be included in the priority list of risk factors. This cut-off was decided on to allow the inclusion of at least one risk factor from each of the six target areas.

After the results of group session 1 were presented in plenary session 1, the research team consolidated the results from all four groups, created an overall priority list that applied to both ABI and SCI, and subsequently formulated so-called “How to” statements for the top risk factors on the priority list (see Table 2). “How to” statements reflect a lateral thinking technique that helps participants to look at problems in a solution-oriented way. For example, if the problem is a lack of a wheelchair-adapted workplace, the “How to” statement would be “How can we adapt the workplace for wheelchair-users”. Such statements were expected to stimulate the development of potential interventions to address the selected risk factors (33).

**Table 2:** Risk factors selected during the condition-specific first group session.

Main topic areas	Risk factor	Vote SCI	Vote ABI	Total
Rehabilitation and Integration	1. Prompt, interdisciplinary diagnostics as the basis for targeted rehabilitation	2	4	6
	2. Interdisciplinary coordination of interventions across disciplines and settings (including employer)	6	5	11
The person and his or her environment	3. Support of injured worker with health issues (physical, cognitive, mental) to retain long-term sustainable employment	9	4	13
The work environment	4. Raise employer and staff awareness about the impact of brain or spinal cord injuries on their injured employees and what to do about it	4	8	8
	5. Limited supply of part-time positions and with flexible working hours on the job market		3	7
Work performance	6. A mismatch in performance expectations between the individual and the employer	3	4	7
	7. Excessive use of own resources in order to cope with the work	4	3	7
Social insurances and other payers	None			
Institutional and professional support	8. Lack of contact person for work-related problems for the worker and the employer in the long-term	8	1	9
	9. Fragmented, poorly networked therapy and integration services, poor communication, especially in the long-term	3	6	9

In the second and third group sessions, the participants were redistributed into four new groups that comprised a mix of participants with ABI and SCI expertise. This remixing provided the participants the opportunity to learn from each other. The participants were asked to formulate possible ways how they could address each “How to” statement, considering not only their own experience and field of expertise but also potential involvement of stakeholders across all levels of the healthcare, social and economic system, i.e., from the patient care micro-level to the provider/hospital meso-level to the policy macro-level. Half of the statements were addressed in group session 2 and the other half in group session 3. The groups presented their results in plenary session 2. Lastly, each participant was asked in plenary session 2 to select the four most relevant interventions by marking them with colored dots (red dots for SCI experts, green dots for ABI experts and yellow dots for persons with non-condition-specific expertise). The participants were prompted to consider the expected impact and feasibility of implementing the intervention when making their selection.

After the conference, the research team summarized the interventions proposed to address the “How to” statements and drafted a summary report detailing the results of plenary session 2. One week after the conference, the summary report was sent to the conference participants and to persons who declined participation in the conference but expressed an interest in being involved in continued activities.

### 2.3. Survey

Three weeks after the conference, a link to a web-based survey was sent to the conference participants and the aforementioned interested persons (Table 1) to enable these stakeholders an opportunity to provide their (final) feedback on the priority interventions resulting from the exercise in plenary session 2, as well as to gage the commitment of individual stakeholders to be involved in implementation activities. In the survey we asked the participants to evaluate the proposed interventions. Since the intervention proposals were written in different styles, some with detail and some with less detail, the research team developed “intervention statements” that harmonized the intervention proposals. Moreover, statements that reflect (1) the potential effectiveness of the respective intervention and (2) the feasibility of implementing the respective intervention, were also formulated. For each statement (i.e., intervention, effectiveness and feasibility), participants were asked the extent of which they agree with the intervention statement, and the statements regarding effectiveness and of feasibility. The questions were rated based on a four-point scale from “agree” to “disagree” with an option of “do not know”. See Table 3.

**Table 3:** Results of the survey. The table shows the absolute number of participants indicating their agreement with the given statements and the corresponding percentage.

Participants were asked the extent they agreed with each of the following intervention, effectiveness and feasibility statements:	Conference participants						Not-conference participants					
		Agree	Partially agree	Partially disagree	Dis-agree	Do not know		Agree	Partially agree	Partially disagree	Dis-agree	Do not know
1. A lifelong contact person for work-related problems should be ensured if needed.	*N* = 22	16 (72.7%)	3 (1.6%)	2 (9.1)	0	1 (4.6%)	*N* = 9	3 (33.3%)	5 (55.6%)	0	1 (11.1%)	0
I believe that having a lifelong contact person to assist with work-related problems is critical to improving the sustainable employment of persons with brain injury or spinal cord injury. (Effectiveness)	*N* = 22	13 (59.1%)	7 (31.8%)	0	0	2 (9.1%)	*N* = 9	2 (22.2)	6 (66.7%)	0	0	1 (11.1%)
I see a starting point for addressing the need for a lifelong contact person to assist persons with a disability with work-related problems in Switzerland. (Feasibility)	*N* = 22	4 (18.2%)	9 (40.9)	5 (22.7%)	1 (4.6%)	3 (13.6%)	*N* = 9	2 (22.2%)	5 (55.6%)	1 (11.1%)	0	1 (11.1%)
2. Given signs of brain injury in an acute setting, possible consequences should be assessed by a neurological specialist and followed up as needed.	*N* = 22	20 (90.9%)	0	0	0	2 (9.1%)	*N* = 9	7 (77.8)	1 (11.1%)	0	0	1 (11.1%)
I believe that a competent diagnosis of brain injury is critical to improving the sustainable employment of persons with brain injury. (Effectiveness)	*N* = 22	13 (59.1%)	5 (22.7%)	0	0	4 (18.2%)	*N* = 9	6 (66.7)	2 (22.2%)	0	0	1 (11.1%)
I see a potential starting point for ensuring an adequate diagnosis for persons with a brain injury in Switzerland. (Feasibility)	*N* = 22	3 (13.6%)	7 (31.8%)	3 (13.6%)	2 (9.1%)	7 (31.8%)	*N* = 9	4 (44.4)	4 (44.4%)	0	0	1 (11.1%)
3. In the case of a brain injury, functional abilities and limitations should be assessed several times throughout the recovery process. A preliminary final assessment should only be made at an appropriate time once the situation has stabilized.	*N* = 22	15 (68.2%)	4 (18.2%)	0	0	3 (13.6%)	*N* = 8	4 (50%)	3 (37.5%)	0	0	1 (12.5%)
I believe that multiple interdisciplinary assessments of the functional consequences of a brain injury conducted at the right time points is critical for improving the sustainable employment of persons with a brain injury. (Effectiveness)	*N* = 22	12 (54.6%)	6 (27.3%)	0	1 (4.6%)	3 (13.6%)	*N* = 8	4 (50%)	3 (37.5%)	0	0	1 (12.5%)
I see a potential starting point for implementing timely interdisciplinary assessments of functional consequences of brain injury. (Feasibility)	*N* = 20	3 (15%)	6 (30%)	4 (20%)	1 (5%)	6 (30%)	*N* = 8	1 (12.5%)	6 (75%)	0	0	1 (12.5%)
4. Every insurance company, as well as clinics and other institutions that provide occupational services should have staff available who are trained in the topic of work and brain injury and whose focus is on supporting the work integration of persons with a brain injury.	*N* = 21	15 (71.4%)	2 (9.5%)	1 (4.8%)	0	3 (14.3%)	*N* = 9	9 (100%)	0	0	0	0
I believe that training integration and insurance professionals (case management) on the topic of work and brain injury is critical to improving the sustainable employment of persons with a brain injury. (Effectiveness)	*N* = 21	15 (71.4%)	4 (19.1%)	0	0	2 (9.5%)	*N* = 9	8 (88.9)	1 (11.1%)	0	0	0
I see a potential starting point for implementing a training program for integration and insurance professionals (case management) on the topic of work and brain injury. (Feasibility)	*N* = 20	8 (40%)	6 (30%)	3 (15%)	0	3 (15%)	*N* = 9	5 (55.6%)	4 (44.4%)	0	0	0
5. Any mismatch between the performance expectations of the employee and that of the employer should be identified, addressed and corrected by persons in a leadership position as early as possible.	*N* = 22	15 (68.2%)	5 (22.7%)	2 (9.1%)	0	0	*N* = 9	8 (88.9%)	1 (11.1%)	0	0	0
I believe that including expectation management between employers and employees as part of institutional health management would significantly improve the sustainable employment of persons with a disability. (Effectiveness)	*N* = 22	12 (54.6%)	7 (31.8.%)	1 (4.6%)	0	2 (9.1%)	*N* = 9	7 (77.8%)	1 (11.1%)	1 (11.1%)	0	0
I see a potential starting point for implementing expectation management between employers and employees as part of institutional health management. (Feasibility)	*N* = 22	7 (31.8%)	9 (40.9%)	2 (9.1%)	0	4 (18.2%)	*N* = 9	4 (44.4%)	4 (44.4%)	0	1 (11.1%)	0
6. In an employment context, flexible work schedule models should be promoted and offered to all employees where possible.	*N* = 22	18 (81.8%)	3 (13.6%)	1 (4.6%)	0	0	*N* = 9	6 (66.7%)	3 (33.3%)	0	0	0
I believe in promoting flexible work schedules as a key way to improving sustainable employment of persons with a disability. (Effectiveness)	*N* = 22	16 (72.7%)	4 (18.2%)	2 (9.1%)	0	0	*N* = 9	6 (66.7%)	3 (33.3%)	0	0	0
I see a potential starting point for promoting and implementing flexible work schedule models. (Feasibility)	*N* = 22	9 (40.9%)	5 (22.7%)	3 (13.6%)	3 (13.6%)	2 (9.1%)	*N* = 9	3 (33.3%)	6 (66.7%)	0	0	0
7. The topic of disability should be a fixed component of diversity management in companies.	*N* = 22	18 (81.8%)	2 (9.1%)	1 (4.6%)	1 (4.6%)	0	*N* = 9	7 (77.8%)	2 (22.2%)	0	0	0
I believe that integrating the topic of disability in diversity management in companies is critical to improving employment opportunities and the sustainable employment of persons with a disability. (Effectiveness)	*N* = 22	15 (68.2%)	4 (18.2%)	2 (9.1%)	1 (4.6%)	0	*N* = 9	7 (77.8%)	1 (11.1%)	1 (11.1%)	0	0
I see a potential starting point for promoting the topic of disability in diversity management in companies. (Feasibility)	*N* = 22	6 (27.3%)	9 (40.9%)	2 (9.1%)	1 (4.6%)	4 (18.2%)	*N* = 9	4 (44.4%)	3 (33.3%)	2 (22.2%)	0	0
8. Additional employer incentives to hire persons with a disability should be created.	*N* = 22	12 (54.6%)	7 (31.8%)	1 (4.6%)	1 (4.6%)	1 (4.6%)	*N* = 9	8 (88.9%)	1 (11.1%)	0	0	0
I believe that providing additional incentives for employers to hire persons with a disability will significantly improve the sustainable employment of persons with a disability. (Effectiveness)	*N* = 22	9 (40.9%)	8 (36.4%)	3 (13.6%)	0	2 (9.1%)	*N* = 9	6 (66.7%)	3 (33.3%)	0	0	0
I see a potential starting point for creating additional incentives for employers to employ persons with a disability. (Feasibility)	*N* = 22	6 (27.3%)	8 (36.4%)	2 (9.1%)	2 (9.1%)	4 (18.2%)	*N* = 9	4 (44.4%)	3 (33.3%)	1 (11.1%)	0	1 (11.1%)
9. Jobs and training positions should be offered specifically for persons with a disability.	*N* = 22	11 (50%)	6 (27.3%)	1 (4.6%)	1 (4.6%)	3 (13.6%)	*N* = 9	6 (66.7%)	3 (33.3%)	0	0	0
I believe that job openings specific for persons with a disability, will improve their sustainable employment. (Effectiveness)	*N* = 22	11 (50%)	7 (31.8%)	0	1 (4.6%)	3 (13.6%)	*N* = 9	6 (66.7%)	3 (33.3%)	0	0	0
I see a potential starting point for promoting job offers specifically for persons with a disability. (Feasibility)	*N* = 22	5 (22.7%)	8 (36.4%)	0	2 (9.1%)	7 (31.8%)	*N* = 9	4 (44.4%)	4 (44.4%)	1 (11.1%)	0	0
10. In vocational integration programs, interventions should focus on empowering persons with a disability in order to build self-competence and promote self-reflection.	*N* = 22	17 (77.3%)	2 (9.1%)	2 (9.1%)	0	1 (4.6%)	*N* = 9	7 (77.8%)	2 (22.2%)	0	0	0
I believe that the empowerment of persons with a disability is critical for improving the sustainable employment of persons with a disability. (Effectiveness)	*N* = 22	16 (72.7%)	4 (18.2%)	1 (4.6%)	0	1 (4.6%)	*N* = 9	7 (77.8%)	2 (22.2%)	0	0	0
I see a potential starting point for promoting the empowerment of persons with a disability. (Feasibility)	*N* = 22	8 (36.4%)	9 (40.9%)	0	0	5 (22.7%)	*N* = 9	4 (44.4%)	5 (55.6%)	0	0	0

## 3. Results

### 3.1. Participants

Twenty-nine participants attended the consensus conference: 15 persons with expertise in ABI, of which four were persons with ABI and two were employers and 10 persons with expertise in SCI, of which two were persons with SCI. Among the health and vocational professionals, five work in vocational rehabilitation, three as case managers at the statutory accident insurance or at a private insurer. Four health and vocational professionals had experience with both conditions, of which one was also an employer of persons with ABI and SCI. One person was a supported employment expert and one was a diversity management specialist.

Four employers cancelled their participation within two days before the conference due to unexpected work-related reasons, illness and an accident. This resulted in an underrepresentation of employers during the conference. In order to include the employers' point of view, the employers who dropped out were additionally invited to participate in the survey (Table 1).

### 3.2. Group session 1

In group session 1, of the 31 risk factors, nine factors were identified as the most important for sustainable employment, each receiving at least six dots (Table 2). Accordingly, the research team formulated nine “How to” statements to correspond to the nine priority risk factors. One of the nine factors was considered missing in the original list of factors by three groups, thus was added: “Missing contact person for injured worker or employer for insurance and work-related issues in the long-term”. Seven of the nine risk factors were chosen by participants in both the SCI and the ABI groups. The factor “Employer and staff sensitivity to the impact of the condition of the injured worker” was exclusively voted in by the two ABI groups. “Lack of a contact person in the long-term” was voted in unanimously by both SCI groups and one dot came from with ABI expertise. Selected risk factors and the “How to” statements based on them are listed in Table 2.

### 3.3. Group session 2 & 3

The results of group sessions 2 and 3 revealed that in total, 133 interventions were proposed by the four groups for the nine “How to” statements. The number of interventions ranged from eight interventions for the “How to” statements *limited supply of part-time positions and flexible working hours on the job market* as well as *optimizing insurance processes and financing of interventions* to 23 interventions for the “How to” statement *interdisciplinary coordination of interventions across disciplines and settings*. Many of the proposals were similar and differed only in the details.

### 3.4. Plenary session 2

During plenary session 2, the participants in all four condition-mixed groups emphasized that they personally benefited from the diversity of viewpoints and experiences from the other participants and that the discussions in group sessions 2 and 3 was a learning experience. Regarding the exercise in which the participants were asked to mark the four most relevant interventions with colored dots, 28 of the 133 interventions were marked by at least one participant. These 28 interventions can be clustered into three thematic groups. One group addressed the time frame of rehabilitation and work integration. These interventions focused on interprofessional team work during the assessment and diagnostic phase in the acute care and rehabilitation of persons with ABI and on the coordination in vocational rehabilitation and work integration for both conditions. The second group of interventions addressed the period after work integration has ended until retirement – the so-called “work-life period” of sustainable long-term employment. For this work-life period, participants focused on support for empowering the person in the long-term, a lifelong contact person, communication with employer and workplace and getting support from job coaches in the long-term. The third group of interventions focused on the availability of adequate jobs, incentives for employers and the lack of legal requirements for the employment of persons with a disability.

Based on the vote at the end of plenary session 2, a tenth intervention that addresses “the empowerment of the persons with ABI or SCI” was added to the nine interventions that addressed the nine “How to” statements.

### 3.5. Survey

The web-based survey was sent to the 29 conference participants and 13 persons who were unable to attend the consensus conference but interested in being involved (called “non-participants” hereafter).

Twenty-two conference participants and nine non-participants completed the survey. Non-responders were mainly health professionals. The nine non-participants consisted of five employers, two representatives of patient organizations, including a person with ABI, and two representatives of non-governmental organizations engaged in work and disability.

Seventy-one to 91% of the consensus participants agreed with six of the ten intervention statements (Table 3), with the highest agreement (91%) received for statement 2 “Given signs of brain injury in an acute setting, possible consequences should be assessed by a neurological specialist and followed up as needed”. The lowest agreement (50%) was found for the statement 8 “Additional incentives for hiring persons with a disability should be created for employers”. The non-participants, on the other hand, agreed with this statement with 89% agreement. There were other differences in the agreement results between the non-participants and the conference participants. For example, the average percentage of agreement (with “agree”) above 70% is higher among the non-participants (85%) vs. among the conference participants (79%). The non-participants even agreed unanimously with statement 4 “Every insurance company, as well as clinics and other institutions that provide occupational services should have staff available who are trained in the topic of work and brain injury and whose focus is on supporting the work integration of persons with a brain injury”.

When looking at the evaluation of effectiveness, a mixed pattern across the 4-point ratings was found. For example, for statement 2, which received the highest agreement among the conference participants, agreement for the corresponding effectiveness statement dropped to 59%, while the “partial agree” (23%) and the “don’t know” (18%) answers rose from 0% and 9%, respectively. Although the conference participants considered statement 1 “A lifelong contact person for work-related problems should be ensured if needed” with 73% as relevant, non-conference participants were less convinced with 33% agreement and 56% partial agreement.

As with the agreement rate for the intervention statement, the agreement rate for effectiveness statement 4 regarding the training of professionals on the topic of brain injury and work, remained stable for conference participants (71%) and was even higher for the non-conference participants (89%).

Furthermore, given that the non-participants comprise five employers (thus reflecting the employer standpoint), it is also noteworthy that the agreement with intervention statement 5 “Any mismatch between the performance expectations of the employee and that of the employer should be identified, addressed and corrected by persons in a leadership position as early as possible” among the non-participants (89%) and its corresponding effectiveness statement (78%) was higher than among the conference participants (68% and 55%, respectively). The employer's standpoint was also clear in the finding that non-participants agreed significantly higher (89%) with intervention statement 8 regarding incentives for employers than the conference participants (55%).

In terms of feasibility, the agreement rate fell dramatically for all statements. Of all statements, statement 2 regarding the neurological assessment and follow up of consequences of a brain injury, that reached 91% agreement among the conference participants, reached the lowest feasibility value of 14% among the conference participants. The non-participants were slightly less pessimistic, with an agreement rate of 44%; this was a drop of only 33 percentage points compared to 77 percentage points of the conference participants. The overall lowest feasibility value (13%) was found among the non-participants for statement 3 that calls for the multiple interdisciplinary assessments of functioning of persons with ABI, while the overall highest feasibility value (56%) was found for statement 4, also among the non-participants. Although still not high, this agreement rate for feasibility statement 4, i.e., training integration and insurance professionals on the topics of work and brain injury, is consistent with the high agreement ratings received for the corresponding intervention (100%) and effectiveness statements (89%).

## 4. Discussion

### 4.1. Summary of results

In this consensus study (consensus conference and survey), diverse stakeholders representing accumulated knowledge and direct experience with sustainable employment of persons with ABI or SCI, discussed and identified the most important risk factors impacting sustainable employment of persons with ABI or SCI and proposed interventions to address them. In the survey, the participants (some of the same stakeholders from the conference as well as additional ones) evaluated the potential effectiveness and feasibility of the proposed interventions.

### 4.2. Most relevant findings

To our knowledge, this is the first attempt to involve microlevel stakeholders with diverse perspectives to prioritize risk factors and identify interventions that address these factors and support sustainable long-term employment of persons with ABI or SCI.

#### 4.2.1. Commonalities and differences

Bringing together stakeholders with an ABI or SCI focus enabled us to learn about health condition-specific problem areas and intervention needs as well as about commonalities experienced by persons living with these two health conditions. The fact that seven of the nine prioritized risk factors were voted in by both health conditions show that there are more commonalities than differences. With regard to interventions, stakeholders with focus on both SCI and ABI supported an intervention that addressed the need for a contact person for work-related problems. However, there are still differences. For example, interventions corresponding to the risk factor that addresses rehabilitation were voted in with specification only for persons with ABI. This may be due to the fact, that in Switzerland, there are four specialized SCI centers which enable a structured and comprehensive continuum of care from acute treatment to work reintegration for persons with SCI. Such specialized centers do not exist for persons with ABI in Switzerland.

#### 4.2.2. Empowerment of injured workers

There was extensive discussion about employers and staff are aware about disability-specific needs of injured workers and protecting injured workers from excessive use of personal resources (time and energy) to handle work demands. During the discussion, some participations with an SCI focus highlighted the need to empower injured workers to advocate for themselves in the work context. However, other participants felt that this places too much responsibility on the employee. Empowering the injured worker aims to enable the person to independently manage critical situations at the workplace. Placing more responsibility on the injured worker was supported in the conference by persons with ABI and SCI as well as the employers, while health professionals, especially those working with persons with ABI, were hesitant to place all the responsibility on the person with ABI. As a safeguard against overburdening the person, the conference participants supported the provision of a lifelong contact person. The experience of participants with an SCI focus underscored the importance of having a lifelong contact person. Such an approach was seen by the representatives of the ABI patient organization as a potential model for facilitating sustainable employment in the future.

#### 4.2.3. The big picture

To ensure that identifying the most important risk factors and the interventions to address them was not vain, we also examined the potential feasibility of implementing the interventions. The results show that the conference participants and survey participants (non-participants) viewed the feasibility of implementing the interventions relatively low. Nevertheless, the interventions addressing flexible work-time-models and the integration of disability in diversity management in companies seem promising. Overall more optimism was shown by the non-participants. For example, 44% of the non-participants agreed that offering jobs specifically for persons with disability is feasible compared to 23% of the conference participants. Although not a strong agreement in terms of percentage, the fact that this result reflects the view of four employers who participated in the survey brings weight to the result. The higher number of employers who participated in the survey (*N* = 4) vs. the conference (*N* = 2) point to the potential influence of decision-makers like employers. During the conference, participants, specifically the case managers at insurance companies and clinicians, emphasized that interventions that involve the implementation of measures in clinics or at insurers, or changes in social legislation require the involvement of decision-makers at the meso- and macro-level.

Although the study was conducted in the Swiss context, several findings may also be applicable beyond Switzerland. First involving micro-level stakeholders with diverse perspectives proved to be a valuable method to identify core problems that directly threaten the sustainable employment of persons with ABI or SCI. Although this multi-stakeholder approach may reveal country-specific risk factors that require country-specific interventions, the approach itself is universal and applicable in any context. To promote a broad understanding of barriers and facilitators of sustainable employment of persons with ABI and SCI beyond country borders, we encourage conducting a study similar to the one presented in this paper in other countries.

### 4.3. Strengths and limitations

As previously mentioned, the present project is likely the first application of a micro-level and multi-level approach to determine a priority list of risk factors to sustainable employment of persons with SCI and ABI and to identify concrete interventions to address each risk factor. Not only is this approach action-oriented, the involvement of stakeholders at the micro-level fosters sustainable work by people with ABI or SCI at the direct person level. Furthermore, the prioritized list provides an important cost-benefit basis for decision-makers at the meso hospital level and macro policy level to revise services and legislation in a targeted way.

This study also has some limitations that may have influenced the results of the consensus process. First, employers were underrepresented during the conference due to last-minute work and health-related cancellations. To offset this underrepresentation of the employers' perspective in the final results, we invited those who cancelled their conference participation as well as other employers to participate in the survey. In the end, the four employers who were unable to attend the conference completed the survey. Secondly, the selection of participants that included persons who had already participated in the preliminary studies and persons new to the topic of sustainable employment could also have biased the discussions. In the end, the mix of different stakeholder experiences and perspectives proved to be stimulating for both groups according to participant comments.

### 4.4. Further research

In addition to promoting further exploration of sustainable employment using the present micro-level and multi-stakeholder approach, further research could involve a broader group of stakeholders that include other health professionals, service providers, payers, experts in disability management, social security offices and the labor market as well as trade unions. Such a far-reaching approach encompassing micro-, meso- and macro-level stakeholders may provide an explanation for why the feasibility of the interventions were seen as low despite the high ratings for relevance. Furthermore, gathering input and data especially from macro-level stakeholders, such as social security administration representatives, policy-makers and economic leaders could be useful in evaluating the impact of potential changes in legislation or labour practices.

Lastly, the methodological approach employed in this study may also be applicable to research on sustainable employment of persons with other health conditions and persons with disability in general.

## 5. Conclusion

Involving micro-level stakeholders with diverse perspectives, such as the persons with the health condition, employers, and health and vocational professionals directly involved in work integration and sustainable employment, is a valuable method for identifying core problems directly threatening the sustainable long-term employment of persons with ABI or SCI. As most of the interventions proposed in this study addressed service needs, successful implementation of these interventions requires the development of guidelines at the payer level as well as legislation that target vocational inclusion of persons with disability. For this and to jump-start the implementation process, stakeholders at the meso- and macro-level have to be involved.

## Data Availability

The datasets presented in this article are not readily available in order to protect the anonymity of participants. Requests to access the datasets should be directed to MF, monika.finger@paraplegie.ch.
