# Supplementary material for: Prioritizing risk factors and identifying target areas to address with interventions to improve sustainable employment of persons with a brain injury or a spinal cord injury – A multi-stakeholder consensus process
Source: Front Rehabil Sci. 2023 Feb 17;4:1049182. doi: 10.3389/fresc.2023.1049182 (PMC9982110; doi:10.3389/fresc.2023.1049182)
Supplement: Supplementary file 1 [file Table1.docx]

# Work sheet: Risk factors for sustained employment of persons with brain or spinal cord injury.

#

**Additional risk factors:** Add risk factors that are still missing from your perspective.

**Evaluation of risk factors:** Put an x if you think a risk factor is very important / significant and you want us to look more deeply into possible solutions for dealing with this risk factor.

| 1. **Main topic area "Rehabilitation and Integration"** | **Evaluation**  (x = significant) |
| --- | --- |
| - 1. Prompt, competent, interdisciplinary diagnostics as the basis for targeted rehabilitation. |  |
| - 1. Interdisciplinary coordination of interventions across disciplines and settings (including employer). |  |
| - 1. Self- and external overload of the person during professional integration |  |
| *Further risk factors on the subject of "Rehabilitation and integration"?* |  |

| 1. **Main topic area "Employees with a disability and their social environment"** | **Evaluation**  (x = significant) |
| --- | --- |
| - 1. Overload due to high work demands - too little time for health management. |  |
| - 1. Lack of competence "in one's own cause". Not being able to represent one's own needs and possibilities and to claim rights. |  |
| - 1. Overload or changes in personal environment: divorce, death of relatives, financial worries, conflicts. |  |
| - 1. Signs at work of impending job loss: accumulation of errors, increasing sick days, withdrawal from social activities. |  |
| - 1. Work is no longer an important part of the person's life plan. |  |
| - 1. **Persons with brain injury:** health problems, such as fatigue, headache, psychological stress. Deterioration of cognitive performance with age. |  |
| - 1. **Persons with spinal cord injury:** health problems and physical overload, such as decubiti, infections, increasing pain, general deterioration of physical performance with age. |  |
| *Further risk factors on the subject of "Employees with a disability and their social environment"?* |  |

| 1. **Main topic area "Work environment":** | **Evaluation**  (x = significant) |
| --- | --- |
| ***Changes in adapted / functioning structures in the working environment*** |  |
| - 1. Change of superiors, new employees/colleagues (values, attitude, loss of knowledge, unrealistic expectations, fear of contact). |  |
| - 1. Economic environment, cost pressure, closure of the business (rationalization of the workplace). |  |
| - 1. Accessibility of the workplace (accessibility by public transport or car, length of commute). |  |
| - 1. **Persons with a brain injury:** reorganization of the department or company or the introduction of new work tools (new work processes, computer programs, machines, vehicles, etc.). |  |
| - 1. **Persons with brain injury or spinal cord injury:** lack of sensitivity of employees/colleagues in dealing with consequences of the disease (lack of tolerance for fluctuations in performance, need for breaks, personality changes). |  |
| - 1. **Persons with a spinal cord injury:** relocation of the company to inaccessible premises, no disabled parking spaces, inadequate infrastructure in interim solutions. |  |
| - 1. **Persons with a spinal cord injury:** reduced career opportunities (inside and outside the company) – dissatisfaction with work. |  |
| ***When changing jobs / looking for a job*** |  |
| - 1. Limited supply of part-time positions and / or flexible working hours on the job market. |  |
| - 1. Discriminating recruiting process (Selection criteria) |  |
| - 1. Concerns and assumptions of employer (additional financial, organizational and administrative burden, lower performance of worker, inadequate job fit, additional costs for infrastructure accommodations) |  |
| *Further risk factors on the subject of "Work environment"?* |  |

| 1. **Main topic area "Work performance"** | **Evaluation**  (x = significant) |
| --- | --- |
| - 1. Mismatch in performance expectations between the person and the employer. |  |
| - 1. Increasing excessive use of own resources in order to cope with the work and expected performance (e.g. increased need for recreation, less time for activities outside of work). |  |
| - 1. **Persons with a brain injury**: Different perception of performance (self-perception – external perception). |  |
| - 1. **Persons with a spinal cord injury:** Affected individuals feel that they have to work harder to be recognized as equal. |  |
| *Further risk factors on the subject of "Work performance"?* |  |

| 1. **Main topic area "Social security and payers"** | **Evaluation**  (x = significant) |
| --- | --- |
| - 1. Pending decisions on pension claims and assumption of costs for benefits in kind, possibly with open court proceedings. |  |
| - 1. Emotional stress due to pending regular pension revisions or sought pension revisions due to a change in work performance. |  |
| *Further risk factors on the subject of "Social security and payers"?* |  |

| 1. **Main topic area "** **Institutional / professional support "** | **Evaluation**  (x = significant) |
| --- | --- |
| - 1. Incomplete or incorrect information by consulting professionals. |  |
| - 1. Lack of knowledge about existing support services. |  |
| - 1. Fragmented, poorly networked therapy and integration services, poor communication, especially in the long-term. |  |
| - 1. Lack of human and time resources of counseling persons and services. |  |
| - 1. Lack of clarity about or funding for professional services. |  |
| *Further risk factors on the subject of "Institutional /professional support"?* |  |
